# Cytomorphological spectrum of thyroid lesions using the Bethesda system: A retrospective study

**DOI:** 10.6026/973206300223453

**Published:** 2026-06-30

**Authors:** Akanksha Awasthi, Shruti Semwal, Sakshi Chaurasia

**Affiliations:** 1 Department of Pathology, Government Medical College, Satna, Madhya Pradesh, India

**Keywords:** Thyroid lesions, fine needle aspiration cytology (FNAC), Bethesda system, cytomorphology, thyroid nodules

## Abstract

Fine Needle Aspiration Cytology (FNAC) is a reliable, minimally invasive technique for evaluating thyroid lesions and the Bethesda System standardizes cytological reporting and clinical decision-making. This retrospective study analyzed 105 patients with palpable thyroid swellings over 1.5 years, classifying lesions according to the Bethesda System (Categories I-VI). The majority of patients were females (86%) with peak incidence in the 31-40 years age group. Most cases were benign (Category II), accounting for 88.5%, while 11.5% were categorized as follicular neoplasm or suspicious for malignancy, indicating higher malignancy risk. Thus, data shows the effectiveness of FNAC combined with the Bethesda system in ensuring accurate diagnosis, risk stratification and appropriate management of thyroid lesions.

## Background:

Thyroid disorders represent a significant global health burden and are reported as the most common endocrine condition encountered in clinical practice worldwide [1]. The epidemiology of thyroid diseases varies significantly between different populations, often influenced by environmental factors such as iodine intake [2]. Recent data from Indian registries indicates that while the majority of these lesions are benign, the incidence of thyroid malignancy is gradually increasing in urban populations [3]. Demographically, thyroid disorders show a distinct predilection for the female gender, often affecting women 4 to 5 times more frequently than men [4]. These disorders can occur at any age but are most commonly observed in the third to fifth decades [5]. Clinically, thyroid swellings present as either a diffuse enlargement of the gland or as discrete nodules, clinically termed as Solitary Thyroid Nodules (STN) [6]. Fine Needle Aspiration Cytology (FNAC) has emerged as the first-line diagnostic modality for the evaluation of thyroid nodules due to its simplicity, safety, cost-effectiveness and high diagnostic accuracy [7]. FNAC plays a pivotal role in distinguishing benign lesions from malignant ones, thereby significantly reducing unnecessary surgical interventions [8]. Despite its widespread use, lack of uniform reporting terminology in the past resulted in ambiguity and inconsistency in clinical decision-making. To address these issues, the National Cancer Institute (NCI) introduced The Bethesda System for Reporting Thyroid Cytopathology (TBSRTC) in 2007 [9]. This system standardized thyroid cytology reporting into six diagnostic categories, each associated with an implied risk of malignancy and recommended clinical management [10]. The Bethesda system has greatly improved communication between cytopathologists and clinicians and has facilitated better patient care [11]. Therefore, it is of interest to evaluate the cytomorphological spectrum of thyroid lesions using fine needle aspiration cytology and classify them according to the Bethesda System, while analysing their distribution across age and gender and determining the proportion of benign and suspicious or malignant cases.

## Methodology:

A study was conducted in the Department of Pathology, Government Medical College, Satna, Madhya Pradesh, India, from April 2024 to October 2025. Total 105 patients of all age groups and both sexes presenting with palpable thyroid swelling underwent FNAC were included.

## Inclusion criteria:

[1] Patients with palpable thyroid swelling

[2] Adequate FNAC smears for evaluation

## Exclusion criteria:

[1] Patients without palpable thyroid swelling

[2] Inadequate or acellular smears (unless otherwise categorized as Bethesda Category I)

## Procedure:

FNAC was performed under aseptic precautions using a 25-gauge needle attached to a 10 ml syringe. Air-dried smears were stained with Giemsa stain and alcohol-fixed smears with Papanicolaou stain. Smears were examined by experienced cytopathologists.

## Cytological evaluation:

All smears were classified into Bethesda categories I to VI as per TBSRTC guidelines.

## Statistical analysis:

Data were entered in Microsoft Excel and analysed. Results were expressed as frequencies and percentages for categorical variables.

## Results:

We studied total 105 cases of thyroid swellings who were subjected to cytological evaluation were enrolled in this study. The patient's age ranged from 11-80 years, with a mean age of years. Out of the total cases, majority (41%) of cases were in the age group of 31-40 years, followed by (22.9%) cases in 41-50 years of age. Among the total cases, majority (n=90, 86%) of the cases were female and 15 (14%) of the cases were male, making the female-to-male ratio of 6:1 (Table 1). Among 105 cases of palpable thyroid swelling present, majority of the cases presented with hypothyroid symptoms (n=40, 38.1%), 12 (11.45%) cases presented with difficulty in swallowing and 8(7.6%) cases were presented with pain (Table 2). In the present study, all 105 cases were classified according to The Bethesda System for Reporting Thyroid Cytopathology (TBSRTC), majority of cases (n=93, 88.5%) belonged to Category II (Benign). Category IV (Follicular Neoplasm) accounted for 7 cases (6.7%), Category III 2 (1.9%) cases while Category V (Suspicious for Malignancy) was observed in 3 cases (2.9%). No cases were categorized under Category I (Non diagnostic), Category III (AUS/FLUS), or Category VI (Malignant) in this study series (Table 3). Consequently, the total number of benign cases was 93 (88.5%), whereas the total burden of follicular and suspicious of malignant cases was 10 (11.5%) (Table 4).

## Discussion:

Fine-needle aspiration cytology (FNAC) remains the cornerstone investigation for the evaluation of thyroid nodules because of its simplicity, cost-effectiveness, rapid turnaround time and high diagnostic accuracy. The introduction of the Bethesda System for Reporting Thyroid Cytopathology (TBSRTC) has significantly improved the standardization of thyroid cytology reporting by providing uniform diagnostic categories linked with estimated risks of malignancy and recommended clinical management strategies. The widespread adoption of this system has facilitated effective communication between cytopathologists, endocrinologists and surgeons, thereby enhancing patient care. In most retrospective studies utilizing the Bethesda system, benign lesions (Category II) constitute the largest proportion of thyroid FNAC diagnoses. Mondal *et al.* (2013) [12] evaluated thyroid aspirates using the Bethesda system and reported that the majority of lesions belonged to the benign category, with colloid goiter being the most frequent diagnosis. Their findings highlighted the usefulness of TBSRTC in reducing unnecessary thyroid surgeries while maintaining diagnostic reliability. Similarly, Mufti and Molah (2012) [13] analyzed thyroid FNAC specimens over a five-year period and demonstrated that benign lesions represented the predominant cytological category. The study further confirmed that the risk of malignancy increased progressively across Bethesda categories, reaching 100% in cytologically malignant lesions. These observations validated the predictive value of the Bethesda classification system and emphasized its role in clinical decision-making. The risk stratification capability of the Bethesda system was further supported by Jo *et al.* (2010), [14] who investigated malignancy rates across Bethesda categories and found a strong correlation between cytological interpretation and final histopathological diagnosis. Their study demonstrated that the malignancy risk rose substantially from benign lesions to suspicious and malignant categories, reinforcing the prognostic significance of Bethesda reporting. Theoharis *et al.* (2009) [15] evaluated the first-year implementation of the Bethesda classification in an academic institution and observed improved reproducibility and clarity in thyroid cytology reporting. The authors reported that standardized terminology facilitated better patient management and reduced ambiguity in indeterminate lesions, particularly within the atypia of undetermined significance and follicular lesion of undetermined significance (AUS/FLUS) category. Another large-scale study by Yang *et al.* (2007) [16] involving thousands of thyroid nodules demonstrated the diagnostic accuracy of FNAC when correlated with histopathological outcomes. Their findings showed that papillary thyroid carcinoma was the most common malignancy identified and highlighted the value of cytological assessment in distinguishing benign from malignant thyroid lesions. The study provided substantial evidence supporting FNAC as the primary diagnostic modality for thyroid nodules. Across the reviewed studies, a consistent cytomorphological pattern emerges in which benign lesions, particularly colloid goiter and lymphocytic thyroiditis; predominate among thyroid aspirates, while papillary thyroid carcinoma represents the most frequent malignant diagnosis which are consistent with findings of Bajaj and Mulukutla (2023) [17] and Kannaujia *et al.* [18]. Female patients are affected considerably more often than males, reflecting the known epidemiological distribution of thyroid disorders. Furthermore, the Bethesda system has proven effective in categorizing indeterminate lesions and guiding the need for repeat FNAC, molecular testing, clinical follow-up, or surgical intervention. Overall, the available literature demonstrates that the Bethesda system is a highly reliable framework for reporting thyroid cytology. Its standardized approach improves diagnostic consistency, facilitates communication among healthcare providers and provides valuable risk stratification that supports evidence-based management of patients with thyroid nodules.

## Conclusion:

Most thyroid lesions were benign and predominantly affected females in the third and fourth decades of life. Bethesda-based reporting enabled effective risk stratification and clinical decision-making. These findings further support FNAC as a reliable and cost-effective first-line diagnostic tool for thyroid nodules.

## Figures and Tables

**Table 1:** Demographic features of the cases studied (N=105)

**Demographic features**		**No. of cases (%)**
Age group	<=20	4 (3.8)
	21-30	20(19%)
	31-40	43(41%)
	41-50	24(22.9%)
	51-60	8(7.6%)
	61-70	5(4.8%)
	<=70	1(1%)
Gender	Male	15 (14%)
	Female	90 (86%)

**Table 2:** Clinical presentation of the cases studied (N=105)

**Clinical presentation**	**No. of cases (%)**
Swelling +	105 (100)
Pain	8 (7.6)
Difficulty in swallowing	12 (11.45)
Hypothyroid symptoms	40 (38.1)

**Table 3:** Bethesda Category Distribution of the cases studied (N=105)

**Bethesda Category**	**Cases**
Category I (Nondiagnostic)	0
Category II (Benign)	93
Category III (AUS/FLUS)	2
Category IV (Follicular Neoplasm)	7
Category V (Suspicious for Malignancy)	3
Category VI (Malignant)	0
Total	105

**Table 4:** Distribution of Cases by Category and Diagnosis (N=105)

**Category**	**Cytological Diagnosis (No. of cases)**	**Total Cases**
Benign	Benign follicular nodular disease (48)	93
(Bethesda Category II)	Colloid Goitre (27)	
	Chronic Lymphocytic Thyroiditis (18)	
	Hashimoto Thyroiditis (7)	
	Thyroglossal cyst (3)	
AUS/Follicular/Suspicious of Malignancy (Bethesda Category III, IV & V)	Follicular neoplasm (7)	12
	Suspicious of malignancy (2)	
	Suspicious for Medullary Carcinoma thyroid (1)	
